# Empirical evaluation of the inter-relationship of articular elements involved in the pathoanatomy of knee osteoarthritis using Magnetic Resonance Imaging

**DOI:** 10.1186/1471-2474-10-133

**Published:** 2009-10-29

**Authors:** Dennis S Meredith, Elena Losina, Gesa Neumann, Hiroshi Yoshioka, Philipp K Lang, Jeffrey N Katz

**Affiliations:** 1 The Section of Clinical Sciences, Division of Rheumatology, Immunology and Allergy, Brigham and Women's Hospital, Boston MA, USA; 2 Department of Radiology, Brigham and Women's Hospital, Boston MA, USA; 3 Department of Orthopedic Surgery, Hospital for Special Surgery, New York NY, USA; 4 Department of Biostatistics, Boston University School of Public Health, Boston MA, USA; 5 Department of Radiological Services, University of California Irvine, Irvine CA, USA

## Abstract

**Background:**

In this cross-sectional study, we conducted a comprehensive assessment of all articular elements that could be measured using knee MRI. We assessed the association of pathological change in multiple articular structures involved in the pathoanatomy of osteoarthritis.

**Methods:**

Knee MRI scans from patients over 45 years old were assessed using a semi-quantitative knee MRI assessment form. The form included six distinct elements: cartilage, bone marrow lesions, osteophytes, subchondral sclerosis, joint effusion and synovitis. Each type of pathology was graded using an ordinal scale with a value of zero indicating no pathology and higher values indicating increasingly severe levels of pathology. The principal dependent variable for comparison was the mean cartilage disease score (CDS), which captured the aggregate extent of involvement of articular cartilage. The distribution of CDS was compared to the individual and cumulative distributions of each articular element using the Chi-squared test. The correlations between pathological change in the various articular structures were assessed in a Spearman correlation table.

**Results:**

Data from 140 patients were available for review. The cohort had a median age of 61 years (range 45-89) and was 61% female. The cohort included a wide spectrum of OA severity. Our analysis showed a statistically significant trend towards pathological change involving more articular elements as CDS worsened (p-value for trend < 0.0001). Comparison of CDS to change in the severity of pathology of individual articular elements showed statistically significant trends towards more severe pathology as CDS worsened for osteophytes (p-value for trend < 0.0001), bone marrow lesions (p = 0.0003), and subchondral sclerosis (p = 0.009), but not joint effusion or synovitis. There was a moderate correlation between cartilage damage, osteophytes and BMLs as well as a moderate correlation between joint effusion and synovitis. However, cartilage damage and osteophytes were only weakly associated with synovitis or joint effusion.

**Conclusion:**

Our results support an inter-relationship of multiple articular elements in the pathoanatomy of knee OA. Prospective studies of OA pathogenesis in humans are needed to correlate these findings to clinically relevant outcomes such as pain and function.

## Background

The pathogenesis of osteoarthritis (OA) was traditionally thought to be a consequence of aging characterized by cartilage degeneration and bony remodeling in the affected joint [1]. However, recent research findings from both in vivo and in vitro human experiments as well as animal models suggest that OA results from the interplay of multiple factors including local inflammation, joint stability, joint loading, alignment and genetic predisposition [2-5]. Together these studies suggest a more contemporary and encompassing view of OA pathogenesis that incorporates all of the articular structures into an integrated process involving the entire joint [6,7].

Diagnostic imaging has played an important role in the study of OA pathogenesis. Radiographs have long served as the standard imaging study for assessing OA [8]. Systems for evaluating radiographic OA such as that of Kellgren and Lawrence focus on osteophytosis and joint space narrowing [9]. Joint space narrowing is an indirect measure of articular cartilage loss [10-12]. Cartilage loss is associated with subchondral sclerosis, a process of subchondral bony remodeling that alters relative bone density. Cartilage loss has also been associated with osteophytosis, the growth of endochondral bone that is thought to be an attempt to stabilize the diseased joint [13]. The use of magnetic resonance imaging (MRI) to visualize structures such as the synovium, meniscus and bone marrow that cannot be visualized with plain radiographs has demonstrated that these structures are also affected in the context of OA [14-33].

Many studies have examined individual articular structures and various cellular and biochemical mediators involved in OA. However, there has been less attention focused on use of MRI to evaluate the involvement of a wide range of knee structures in the context of OA. Several scoring systems, most notably the Whole-Organ Magnetic Resonance Imaging Score (WORMS) of the knee and the Boston Leeds Osteoarthritis Knee Score (BLOKS) have presented semi-quantitative forms that attempt to include all of the articular structures visible on MRI in a comprehensive assessment of knee OA [34-36].

In this cross-sectional analysis, we evaluated knee MRI studies from a patient cohort with widely varying degrees of OA. We used a previously reported semi-quantitative rating form to test the whole joint theory of OA pathoanatomy [37]. The form was used to assess cartilage, bone marrow lesions (BMLs), osteophytes, subchondral sclerosis, joint effusion and synovitis. We first hypothesized that the severity of cartilage damage in a given knee will be associated with both the number of articular elements involved and the severity of the changes for each articular element. We selected cartilage damage as our initial common measure of OA severity since cartilage degeneration has been a central element of previous traditional imaging studies and chondrocyte dysfunction has been shown to play a role in the cellular/biochemical aspect OA pathogenesis [38-43]. We then hypothesized correlations in the severity of multiple articular elements independent of their relationship to cartilage damage.

## Methods

### Cohort entry and exclusion criteria

To be eligible for this study, patients must have undergone arthroscopic partial meniscectomy (APM) in 2002 at our institution and must have been at least 45 years old at the time of surgery. APM was used to identify subjects since patients undergoing APM frequently have both OA and a preoperative knee MRI. A chart review of eligible patients was performed. Patients with documented anterior cruciate ligament repair or inflammatory arthritis were excluded. Patients with evidence of partial or complete anterior cruciate tears on MRI were excluded. We also excluded patients who did not have MRI performed at our institution to ensure that the MRI studies were all done in a standardized manner.

### Institutional Review

All research conducted within this manuscript is in compliance with the Helsinki Declaration and was approved by the Partners Healthcare Institutional Review Board.

### MRI scanning protocol

Each MRI examination evaluated in this study was performed according to a standardized institutional protocol. MRI of each knee at 1.5 T consisted of coronal T1 weighted spin echo (TR [relaxation time] 550 ms, TE [excitation time] 20 ms) with 3.5 mm-thick sections, a 0.5 mm intersection gap, 20.83 kHz bandwidth (BW), 2 numbers of excitations (NEX), a 14 cm field of view (FOV), 512 × 256 matrix; sagittal fast spin echo proton density weighted sequences (TR 2400 ms, TE 37 ms) with 3.5 mm-thick sections, a 0.5 mm intersection gap, 32 kHz BW, 2 NEX, a 14 cm FOV, 512 × 256 matrix; sagittal fat saturated fast spin echo proton density weighted sequences (TR 2950 ms, TE 20 ms) with 3.5 mm-thick sections, a 0.5 mm intersection gap, 32 kHz BW, 2 NEX, a 14 cm FOV, 512 × 256 matrix; coronal short tau inversion recovery sequences (TR 3000 ms, TE min Full ms, TI 160 ms) with 4.0 mm-thick sections, a 1.0 mm intersection gap, 31.25 kHz BW, 2 NEX, a 14 cm FOV, 256 × 192 matrix; and axial T2 weighted fast spin echo (TR 3625 ms, TE1/TE2 20/130 ms) with 3.5 mm-thick sections, a 0.5 mm intersection gap, 17.86 kHz BW, 1 NEX, a 14 cm FOV, 256 × 224 matrix. Scans were performed using a dedicated extremity coil.

### MRI Assessment

When scoring the knee, an assessment of the articular structures was made using whichever of the available sequences provided the best information. To avoid potential bias, an independent observer not involved in the care of patients and blinded to the intention of this study evaluated the MRI scans using a standardized semi-quantitative assessment form developed by the investigators. The reader was a medical student trained by two attending musculoskeletal radiologists. The intra-rater reliability for this student was assessed in 28 patients with weighted kappas for cartilage lesion grade, cartilage lesion size, BMLs, osteophytosis and synoviitis. Caritlage lesions were judged over 12 regions, giving 12 weighted kappas. The median of these 12 weighted kappa values for cartilage grade was 0.62 and for cartilage lesion size was 0.56 [44]. Bone marrow lesions were assessed in the medial femoral condyle and medial tibial plateau. The median of these two weighted kappa values was 0.70. Osteophytes were scored for the medial and lateral compartments. The median kappa value was 0.80. Synoviitis was assessed across the entire knee with a kappa value of 0.21.

For this study, a grading form was used to evaluate pathology of six distinct articular elements: cartilage, osteophytes, subchondral sclerosis, BMLs, joint effusion and synovitis. Each type of pathology was graded using an ordinal scale with a value of zero indicating no pathology and higher values indicating increasingly severe levels of pathology. Subchondral sclerosis was similarly noted as present or absent for the medial, lateral and patellofemoral knee compartments. Subchondral sclerosis was defined as an increase the area of low signal intensity on both T1 and T2-weighted images within the subchondral bone. The methodology for scoring the MRI studies and modifications to those scores for the purpose of statistical analysis are described in Table 1. The scales for assessing cartilage and bone marrow lesions were based on previous reports [37,44,45]. Briefly, the size and severity of cartilage lesions were recorded for subdivisions of the medial, lateral and patellofemoral compartments. Lesion severity was graded on a seven level scale ranging from 0 = normal to 6 = full thickness cartilage loss. Of note, fraying was defined as increased signal with superficial linear pattern on fast spin echo proton density weighted or fat saturated fast spin echo proton density weighted sequences. Fissuring was defined as full thickness cartilage defect measuring < 1 mm in size on its largest dimension. Lesion size was graded on a five level scale from 0 = normal to 4 = >3 cm. In cases were multiple lesions, were present, the most severe lesion was scored. Osteophytosis was evaluated in the medial, lateral and patellofemoral compartments. Within each compartment, individual osteophytes were scored from zero to three based on their largest visible dimension (0 = <1 mm to 3 = >5 mm). The scores for all osteophytes in a compartment were totaled and this total score was categorized to yield a composite score from zero to three. For BMLs, the approximate diameter of the lesions was classified using a four level scale (0 = none to 3 = >1.5 cm). The scores for individual lesions were summed for both femoral and tibial surfaces as well as the patellofemoral compartment to give a composite score ranging from none = 0 to severe = 6+. Joint effusion was assessed based on the greatest width of the fluid accumulation perpendicular to the long-axis of the leg (0 = none: 0.0-0.5 cm to 3 = severe: >2.0 cm). Finally, synovitis was assessed based on the number of thickened villi visible on T2-weighted scans (0 = none to 3 = severe: >15 villi with marked thickening).

**Table 1:** MRI Assessment Criteria

**Articular Element**	**MRI Scoring**	**Analysis/Classification**
Cartilage	Two step process for each joint surface:	Cartilage signal summarized as:
	1. Grade degree of cartilage damage:	0-None = normal
	0 = Normal	1-Mild = heterogeneity or fraying
	1 = Increased signal heterogeneity without surface disruption	2-Moderate = fissuring or thinning < 50%
	2 = Fraying (superficial only)	3-Severe = thinning > 50% or full thickness
	3 = Fissuring (full thickness lesions <1 mm in width)	Lesion size summarized as:
	4 = Thinning <50% cartilage thickness	0-None = normal
	5 = Thinning >50% cartilage thickness	1-Mild = < 1 cm
	6 = Full thickness cartilage loss	2-Moderate = 1-2 cm
	2. Score greatest diameter of greatest severity lesion	3-Severe = > 2 cm
	0 = Normal	Cartilage signal and lesion size scores summed over 17 regions to give hypothetical range 0-102.
	1 = <1 cm	None = 0
	2 = 1-2 cm	Mild = 1-30
	3 = 2-3 cm	Moderate = 30-60
	4 = >3 cm	Severe = > 60
Osteophytes	Two step process for each joint compartment:	Compartmental scores (medial, lateral, patellofemoral) were summed for the whole knee. The possible score range was 0-9. Final assessment of osteophytosis:
	1. Grade individual osteophytes according to the size of the greatest dimension:	None = 0
	0 = <1 mm	Mild = 1-3
	1 = 1-2 mm	Moderate = 4-5
	2 = 2-5 mm	Severe = 6+
	3 = >5 mm	
	2. Sum scores of all individual osteophytes to give compartmental score:	
	0 = normal individual sum 0	
	1 = mild individual sum 1-2	
	2 = moderate individual sum 3-4	
	3 = severe individual sum >4	
Bone Marrow Lesions	Greatest diameter of each lesion recorded for both femoral and tibial surfaces as well as the patellofemoral compartment:	Scores for patellofemoral compartment and femoral and tibial surfaces were summed. The possible score range was 0-15. Final assessment of bone marrow lesions:
	0 = None	None = 0
	1 = Mild (<0.5 cm)	Mild = 1-3
	2 = Moderate (0.5-1.5 cm)	Moderate = 4-5
	3 = Severe (>1.5 cm)	Severe = 6+
Effusion	Greatest diameter of the effusion perpendicular to the long-axis of the leg:	Effusion was recorded as a single score for the whole knee. No changes were made for final analysis.
	0 = None (0.0-0.5 cm fluid)	
	1 = Mild (0.5 -- 1.0 cm fluid)	
	2 = Moderate (1.0 -- 2.0 cm fluid)	
	3 = Severe (>2.0 cm fluid)	
Synovitis	Assessed by counting the number of thickened villi seen on T2-weighted sequences	Synovitis was recorded as a single score for the whole knee. No changes were made for final analysis.
	0 = None (<5 villi seen, all normal thickness)	
	1 = Mild (6-10 villi seen, some thickened)	
	2 = Moderate (11-15 villi seen, mostly thickened)	
	3 = Severe (> 15 villi seen, marked thickening)	
Subchondral Sclerosis	For each compartment:	Final score determined by number of the 3 knee compartments with evidence of sclerotic change
	0 = Absent	0 = Absent
	1 = Present	1 = Mild
		2 = Moderate
		3 = Severe

### Statistical Analysis

We developed a cartilage damage score (CDS) to capture the total burden of cartilage involvement. This was calculated as the sum of all cartilage damage severity and lesion size scores across all regions of the knee. First, we created categories of none, mild, moderate and severe for cartilage signal and for cartilage lesion size. These categories are shown in Table 1. These categories were represented numerically in our analysis using a scale from 0-3. We evaluated cartilage in 17 different regions (anterior, middle and posterior thirds of medial femoral, lateral femoral, medial tibial and lateral tibial as well as three patellar regions and two trochlear regions. Cartilage signal and lesion size scores (range 0-3) were summed in each region so that each region had a possible range of 0-6 and the 17 regions had a range of 0-102. Next we divided the overall score into categories of None = 0, Mild = 1-30, Moderate = 31-60 and Severe = > 60. These categories were created on clinical grounds. We reasoned that in mild cases, fewer than half the regions would be involved and the involvement would be mild with respect to cartilage signal and lesion size. The score, given these findings, would be in the range of 1-30. On the other hand, in severe OA, more than half of the 17 regions would be involved and the region scores would be in the moderate to severe range. Thus, the overall score would be over 60.

Within each CDS group, we analyzed the percentage of the total cohort represented by three subgroupings based on the number of articular elements showing pathological changes (0-1 element, 2-3 elements, 4-5 elements). For the purpose of characterizing the severity of pathology within the cohort, we created subgroups based on the severity of pathology for individual articular elements. These groups were formed based on the classifications of the semi-quantitative MRI scoring system. This process is described in Table 1. For example, to assess synovitis or joint effusion, which were scored for the whole knee, no changes were made from the original score of none, mild, moderate or severe. For elements such as osteophytosis and BMLs, which are scored compartmentally, compartmental scores were summed over the whole knee and the resulting composites were divided evenly into four groups representing none, mild, moderate and severe pathology. The chi-squared test for trend was used to make statistical comparisons between the groupings. A Spearman test for non-parametric correlations was used to assess the inter-relationship between worsening levels of pathology for each articular element. All analyses were performed in SAS.

## Results

### Patient recruitment

We identified 216 patients over 45 years old who underwent arthroscopic partial meniscectomy (APM) at our institution in 2002. Of these 140 (65%) had a fully scored MRI performed at our institution and comprised the study cohort. These patients were an average to two years older than those not included. 61% of those included were female as compared with 50% of those not included. Neither of these differences were statistically significant.

### Demographic characteristics of the study cohort

The cohort had a mean age of 61 years (sd 9.5, range 45 to 89). 61% of the cohort was female.

### Distribution of disease severity

The distribution of cartilage disease severity scores in the study cohort is listed in Table 2. 99% of knees had some degree of cartilage signal abnormality in at least one articular surface. The most commonly affected surface was the medial femoral condyle followed by the lateral femoral condyle. All levels of cartilage damage from normal to full thickness defects were represented. 67% of knees had osteophytes. 37% had evidence of bone marrow lesions. 16% had evidence of synovitis. 63% had subchondral sclerosis. 68% had an effusion. All of the knees had meniscal tears.

**Table 2:** Distribution of Disease Severity within the Study Cohort for Individual Articular Elements.

	**Severity of Disease**				
**Articular Element**	**None**	**Mild**	**Moderate**	**Severe**	**Total**

Cartilage	1	46	37	16	100
Osteophytes	33	35	11	21	100
Bone Marrow Lesions	63	31	5	1	100
Effusion	32	36	26	6	100
Synovitis	84	9	6	1	100
Subchondral Sclerosis	37	36	18	9	100

For all articular elements included in this table, the data are represented in terms of percentage of the total cohort having evidence of pathological changes of that type on MRI. Each row represents an individual articular element. Criteria for assessing the severity of each element (Cartilage, Osteophytes, Bone Marrow Lesions, Effusion, Synovitis and Subchondral Sclerosis) are given in Table 1.

### Cartilage damage vs. number of articular elements showing pathological changes

**Figure 1:**
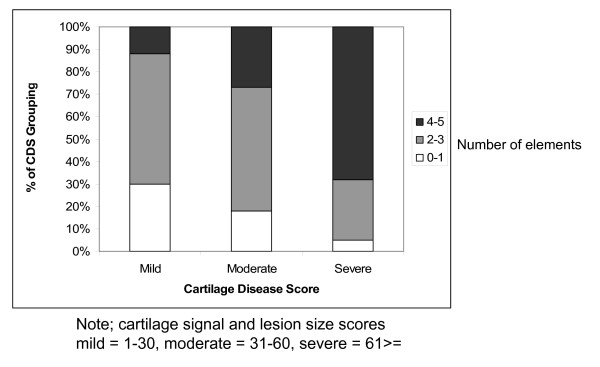
**The number of articular elements showing pathological changes stratified by cartilage disease score (CDS)**. CDS is a summation of all compartmental cartilage disease severity and lesion size scores across the entire knee. These scores were then categorized as mild, moderate and severe based on the methodology described in Table 1. The number of articular elements involved is categorized as 0-1 element, 2-3 elements and 4-5 elements. The y-axis indicates the percentage of patients in that CDS grouping with evidence of pathological change in articular elements on MRI. The articular elements included in this figure are osteophytosis, subchondral sclerosis, bone marrow lesions, joint effusion and synovitis. The trend of increasing number articular elements with increasing CDS is statistically significant (p-value for trend < 0.0001).

To test the whole joint hypothesis for OA, we asked if the number of articular elements showing some pathological change increased in association with worsening severity of cartilage damage. To investigate this question we divided the cohort based on the CDS into groups we termed mild, moderate and severe using the methodology described in Table 1. We formed three sub-groupings based on the number of articular structures with pathological changes in each knee (0-1 affected articular elements; 2-3 such elements; and 4-5 elements). Next, we examined the distribution of this subgrouping of articular structures involved across the CDS groups. The results are shown in Figure 1. This revealed a statistically significant trend in which knees with worse cartilage disease had a greater number of articular elements with pathological changes (p-value for trend < 0.0001).

### Cartilage damage vs. severity of pathological changes for individual articular elements

**Figure 2:**
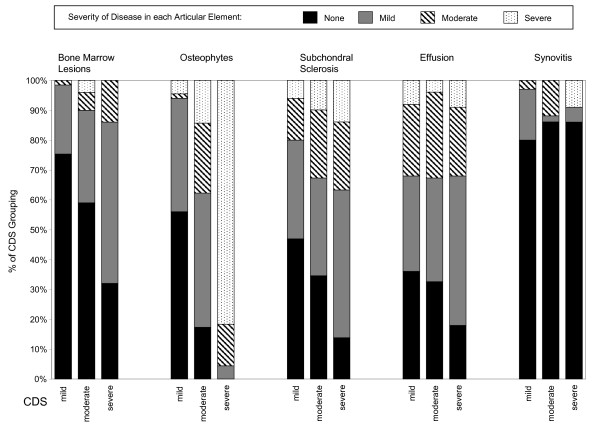
**Association of cartilage degeneration severity with pathology for osteophytes, bone marrow lesions, subchondral sclerosis, joint effusion and synovitis**. There are 5 groupings of CDS scores along the x-axis indicating the articular element being examined. These elements are bone marrow lesions, osteophytosis, subchondral sclerosis, joint effusion and synovitis. For each of these elements, three vertical bars are presented, representing mild, moderate and severe cartilage disease score (CDS). The legend shows sub-groupings based on the severity of disease for an individual articular element. The classification schema for these sub-groupings is described in Table 1. The y-axis indicates the percentage of patients in a CDS grouping with evidence of the severity of pathological change for each sub-grouping. The trend toward greater involvement of each element with greater CDS score is evident for osteophytes (p-value for trend < 0.0001), bone marrow lesions (p = 0.0003) and subchondral sclerosis (p = 0.009), but not synovitis (p = 0.58) or effusion (p = 0.46).

We investigated the association of the severity of disease of individual articular elements with the severity of the cartilage disease score. We again divided the cohort into three groups based on the CDS. Within these groups we created subgroupings based on the severity of disease for individual articular elements. Each of the articular elements included in this analysis-- osteophytes, BMLs, joint effusion, and synovitis-- was graded as none, mild, moderate, or severe except for subchondral sclerosis, which was graded as present or absent. The criteria for these classifications are listed in Table 1. We compared the distribution of these subgroupings across CDS groups. The results are shown in Figure 2. This analysis revealed a statistically significant trend, in which worse CDS scores were associated with worse severity of disease for osteophytes (p < 0.0001), BMLs (p = 0.0003) and subchondral sclerosis (p = 0.009), but not joint effusion or synovitis (p > 0.50 for each).

### Spearman correlation coefficients between individual elements

Finally, we investigated the relationship between individual articular elements using the Spearman non-parametric correlation coefficient. The test assessed the correlation between increasing severity of pathology in each articular element. The results of this analysis are shown in Table 3. Of note, cartilage damage had a moderately strong correlation with BMLs and osteophytes. There was a moderate correlation between joint effusion and synovitis. However, joint effusion and synovitis had only a weak correlation with osteophytes or cartilage damage.

**Table 3:** Spearman correlation coefficients for increasing severity of pathology between articular elements.

	**Cartilage**	**BME**	**Synovitis**	**Effusion**
BME	0.35			
Synovitis	0.04	-0.02		
Effusion	0.05	0.36	0.33	
Osteophytes	0.69	0.27	0.10	0.08

This correlation table shows the Spearman correlation coefficients for increasing levels of pathology between articular elements.

## Discussion

We investigated the inter-relationship of multiple articular elements involved the pathoanatomy of OA in a cohort of 140 patients who had arthroscopic partial meniscectomy and knee MRI at our institution in 2002. We used a semi-quantitative MRI grading form to evaluate each MRI study. The MRI studies were routine, clinical exams using a 1.5 T MRI scanner. The scoring form quantitatively evaluated the severity of individual articular elements including cartilage, osteophytosis, subchondral sclerosis, bone marrow lesion, joint effusion and synovitis. Knees with worse cartilage damage had a greater number of articular elements showing pathological changes. Worse cartilage damage was also associated with greater disease severity for the majority of the articular elements examined. We also examined the correlation between increasing severity of pathology in each articular element. This showed a moderate correlation between cartilage damage, osteophytes and BMLs as well as a moderate correlation between joint effusion and synovitis. However, cartilage damage and osteophytes were only weakly associated with synovitis or joint effusion. These are the first data we are aware of examining the inter-relationship between pathological changes of multiple articular elements in a patient cohort. Our results support the theory that OA pathoanatomy involves most articular elements of the knee.

Our findings are consistent with previously reported data using in vivo and in vitro models of OA pathogenesis. In the following paragraph, we briefly review the inter-relationships of the various articular elements that have been previously reported. The central role of hyaline cartilage loss in the pathogenesis has been well established [38-43]. Joint space narrowing, osteophytosis and subchondral sclerosis have all been shown to be closely associated in the pathogenesis of OA [13,46,47]. A previous study by Hayes et al. has shown a significant association between worsening Kellgren Lawrence grade on knee radiographs and pathology of cartilage, osteophytes, meniscal tears, synovitis, and joint effusion on MRI [48]. Bone marrow lesions (BMLs) have been shown to be strong risk factors for structural deterioration of knee OA [23]. The risk of cartilage deterioration in the setting of BMLs appears to be independent of pre-existing OA [49,50]. The relationship of BMLs to pain is less clear. Felson et al. have shown a strong relationship between the presence of BMLs and knee pain [22]. However, other studies have not shown that changes in the size or number of lesions correlate with changes in WOMAC score [51,52]. Inflammation of the synovium may contribute to the progression of OA through the production of inflammatory cytokines that promote cartilage degradation and alteration of the lubricating properties of the synovial fluid [53,54]. There is some debate as to the optimal MRI sequences for imaging the synovium. Bredella et al. have shown that the proton density and fast spin echo images with fat suppression, such as the ones used in this study, have a high sensitivity and specificity for detecting synovial pathology when compared to arthroscopy [55]. However, some authors favor the use of gadolinium [17]. The assessment of synovium can be based on hypertrophy at selected sites [20] or a comprehensive assessment of synovial volume [16]. Overall moderate to good agreement has been demonstrated between both of these methods of assessment [56].

The use of patients with concomitant meniscal pathology is a potentially confounding factor. Several recent studies have shown that the meniscus is frequently involved in knees with OA. In a large cohort of 245 elderly subjects aged 70-79 with knee OA the prevalence of meniscal lesions was 83% in men and 73% in women [31]. The meniscal abnormalities were strongly associated with cartilage defects. However, the presence of meniscal pathology in the setting of OA is poorly correlated with patient symptoms. One study demonstrated that patients with OA and meniscal tears did not have any more knee pain than patients with OA alone [33]. A recent paper showed that in patients with radiographic evidence of OA, the prevalence of meniscal tears among patients with knee pain was 63% versus 60% among those with no symptoms [30].

The cross-sectional design of this study imposed limitations such as an inability to prospectively follow pathological changes in individual knees over time and an inability to use clinical criteria in the selection of our patient cohort. In particular, the cross-sectional nature of the data makes it impossible to determine which features come first in the sequence of pathogenesis. Thus, we cannot use our data to support or disprove specific pathophysiological mechanisms. Another potential limitation of this study is the use of exact measurements to assess the size of specific articular elements such as cartilage defects, BMLs or osteophytes rather than proportional measurements normalized to patient size. However, this issue is unlikely to distort the findings of our analyses because we do not make specific comparisons across gender or height. The strengths of our study included the use of standardized MRI assessment tool and MRI assessment by a trained observer blinded to the intention of the study.

## Conclusion

Our results support the inter-relationship of multiple articular elements in the pathoanatomy of knee OA. These findings highlight the need for future studies of OA pathogenesis in humans using MRI. Specifically, future studies will need to investigate the correlation of the scoring methods used in this study with clinical symptoms and radiographic progression of OA in a prospective cohort.

## Competing interests

The authors declare that they have no competing interests.

## Authors' contributions

DSM helped to design the study, score MRIs and prepared the manuscript. EL performed all statistical analyses and helped prepare the manuscript. GN and HY created the MRI scoring form and helped to edit the manuscript. PK helped to design the MRI scoring form and edit the manuscript. JNK designed the study, helped perform statistical analyses and prepare the manuscript. All authors have read and approved the final manuscript.

## Pre-publication history

The pre-publication history for this paper can be accessed here:


